# Which heart failure patients benefit most from non-invasive telemedicine? An overview of current evidence and future directions

**DOI:** 10.1007/s12471-024-01886-4

**Published:** 2024-08-14

**Authors:** Jorna van Eijk, Kim Luijken, Jaap Trappenburg, Tiny Jaarsma, Folkert W. Asselbergs

**Affiliations:** 1 Department of Nursing Science, Julius Centre for Health Sciences and Primary Care, University Medical Centre Utrecht, Utrecht, The Netherlands; 2 Julius Centre for Health Sciences and Primary Care, Department of Epidemiology, University Medical Centre Utrecht, Utrecht, The Netherlands; 3 The Healthcare Innovation Centre, Julius Centre for Health Sciences and Primary Care, University Medical Centre Utrecht, Utrecht University, Utrecht, The Netherlands; 4 Department of Health, Medicine and Caring Science, Linköping University, Linköping, Sweden; 5 Department of Cardiology, Amsterdam University Medical Centres, University of Amsterdam, Amsterdam, The Netherlands; 6 Health Data Research UK and Institute of Health Informatics, University College London, London, UK

**Keywords:** Heart failure, Telemedicine, Telemonitoring, eHealth, Personalised medicine, Subgroups

## Abstract

Telemedicine in heart failure (HF) management may positively impact health outcomes, but varied effects in studies hinder guidance in HF guidelines. Evidence on the effectiveness of telemedicine in HF subpopulations is limited. We conducted a scoping review to evaluate and synthesise evidence on the effectiveness of telemedicine across HF subpopulations that could guide telemedicine strategies in routine practice. Meta-analyses concerning randomised controlled trials (RCTs) with subgroup analyses on telemedicine effectives were identified in PubMed. We identified 15 RCTs, encompassing 21 different subgroups based on characteristics of HF patients. Findings varied across studies and no definite evidence was found about which patients benefit most from telemedicine. Subgroup definitions were inconsistent, not always a priori defined and subgroups contained few patients. Some studies found heterogeneous effects of telemedicine on mortality and hospitalisation across subgroups defined by: New York Heart Association (NYHA) classification, previous HF decompensation, implantable device, concurrent depression, time since hospital discharge and duration of HF. Patients represented in the RCTs were mostly male, aged 65–75 years, with HF with reduced ejection fraction and NYHA class II/III. Traditional RCTs have not been able to provide clinicians with guidance; continuous real-world evidence generation could enhance monitoring and identify who benefits from telemedicine.

**Supplementary Information:**

The online version of this article (10.1007/s12471-024-01886-4) contains supplementary material, which is available to authorized users.

## Introduction

Heart failure (HF) is a major public health problem, affecting over 64.3 million people worldwide [1]. This chronic condition with an unpredictable trajectory, an accelerated decline of cardiac function, is characterised by frequent exacerbations often leading to hospitalisation, an increase in symptoms and dependency and risk of death [2]. Hospitalisations of HF patients can be reduced by optimal medical management, self-care (i.e. healthy diet, medication adherence, exercise), education (i.e. information about HF, medical treatment, self-care aspects, living with HF), and adequate monitoring of vital signs and symptoms [2]. Telemedicine is increasingly considered a meaningful intervention to support patients in optimising HF management, self-care support and symptom monitoring to improve care and prevent (re)hospitalisation [3]. It is an umbrella term for a wide range of digital technologies that exchange digital health information between healthcare professional and patient to support and optimise the care process remotely [4].

Numerous meta-analyses have evaluated the clinical and cost-effectiveness of telemedicine in HF patients, regardless of patient subgroups. Overall, these studies hint towards a positive effect of telemedicine on hospital (re)admission, length of stay, mortality and reduced healthcare costs, but with a wide variation in effects between studies [5–7]. As a result, HF guidelines lack specific advice on how, when and for whom telemedicine should be provided. However, at the local level (e.g. hospital) there is somewhat more guidance on telemedicine, which stems particularly from expert consensus [8]. Consequently, telemedicine is implemented in different formats with varying objectives, intervention components and implementation strategies.

**Fig. 1:**
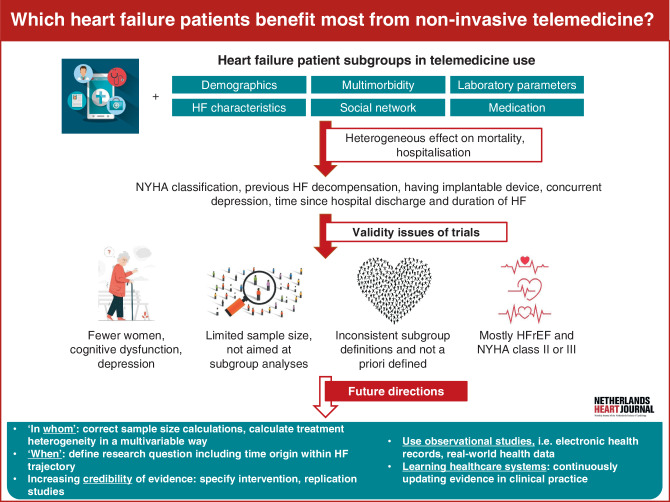
Infographic: Which heart failure patients benefit most from non-invasive telemedicine? An overview of current evidence and future directions. *NYHA* New York Heart Association, *HF* heart failure, *HFrEF* heart failure with reduced ejection fraction

A recently conducted comprehensive meta-analysis examining the clinical effectiveness of telemedicine and its various modalities included studies up to 2022, providing a structured comparison of telemedicine interventions. The findings of this analysis emphasised the importance of future research efforts focusing on defining specific subgroups of patients and corresponding telemedicine modalities [6]. Knowledge on modification of the effectiveness of telemedicine across patient characteristics will contribute to targeting telemedicine to those groups anticipated to benefit most. In this scoping review, we discuss and synthesise existing evidence on the effectiveness of telemedicine across HF subpopulations to guide telemedicine strategies in routine practice (Infographic: Fig. 1).

## Methods

A scoping review of studies reporting on the effectiveness of telemedicine in HF subpopulations was conducted. This approach aims to select literature to map current knowledge, identifying gaps that can guide future studies and innovations [9]. Due to the nature of scoping reviews, PRISMA/PROSPERO guidelines were not appropriate.

### Search

A literature search to identify meta-analyses including randomised controlled trials (RCTs) on telemedicine in HF patients was performed in PubMed, on 13 November 2023, and included full-text references from 2018 to that date, restricted to meta-analyses. Search terms were ‘telemedicine AND heart failure’, ‘telemonitoring AND heart failure’. Title and abstract were screened for articles about clinical effectiveness of non-invasive telemedicine interventions in HF patients. This search strategy resulted in 19 meta-analyses (Electronic Supplementary Material: overview of meta-analyses). The included meta-analyses were used to search for RCTs.

### Study selection

RCTs were collected; duplicates and inaccessible/non-English/non-Dutch full-text articles were removed. Remaining full-text articles underwent reassessment, focusing on subgroup and clinical effectiveness analyses. RCTs were only excluded if they lacked subgroup analyses.

### Data extraction

Data from selected RCTs were extracted and summarised in a table comparing the studies. Potential limitations of the included studies were identified, i.e. small sample size, analyses of primary or secondary aim, different defined outcomes and telemedicine interventions between studies and representativeness of the study sample. We considered these limitations when reporting the findings.

## Results

**Fig. 2:**
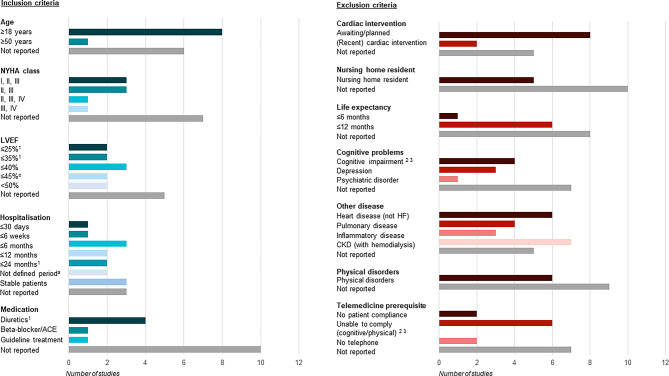
Inclusion and exclusion criteria regarding patient characteristics for randomised controlled trials. *NYHA* *class* New York Heart Association classification, *LVEF* left ventricular ejection fraction, *ACE* angiotensin-converting enzyme, *HF* heart failure, *CKD* chronic kidney disease; ^a^A trial has a combination of three different inclusion criteria in one statement; therefore all criteria were counted separately: diagnosed with an LVEF ≤ 25% measured at least twice within the past 6 months *or* an LVEF ≤ 35% and at least one cardiac decompensation with hospitalisation due to CHF *or* therapy with intravenous diuretics within 24 months prior to enrolment; ^b^A trial has a combination of two different inclusion criteria in one statement; therefore all criteria were counted separately: diagnosed dementia or difficulty in understanding instructions or using the scale; ^c^A trial has a combination of two different inclusion criteria in one statement; therefore all criteria were counted separately: patients who did not have the cognitive or physical ability (dementia, or weight > 204 kg) required to participate fully in the BEAT-HF intervention

Fifteen RCTs on clinical effectiveness of telemedicine were identified in which subgroup analyses were performed [10–24]. Study characteristics and performed subgroup analyses per included RCT are shown in Table S1 (Electronic Supplementary Material). The eligibility criteria of the evaluated RCTs showed that mostly adults recently discharged from HF hospitalisation, having HF with reduced ejection fraction (HFrEF) and New York Heart Association (NYHA) class II/III, are selected for participation (Fig. 2; [10–24]). Patients were not eligible to participate when diagnosed with multiple heart diseases, chronic kidney disease (CKD), cognitive problems (i.e. memory disorders, depression), physical problems, or when living in a nursing home and/or having a life expectancy less than 1 year [10–24]. Correspondingly, baseline characteristics of the evaluated RCTs indicated that most patients were male, aged between 65 and 75 years, with HFrEF and NYHA II/III. A few RCTs mentioned digital skills and having a mobile device as selection criteria for telemedicine [12, 14, 15, 23]. It is unclear whether individuals without digital skills or mobile devices were purposely excluded, or whether data were unavailable or not reported for other reasons. Secondly, some RCTs measured social economic status (SES) at baseline, but no study assessed heterogeneity of telemedicine effectiveness across SES status [10, 11, 14, 18].

### Patient subgroups

**Fig. 3:**
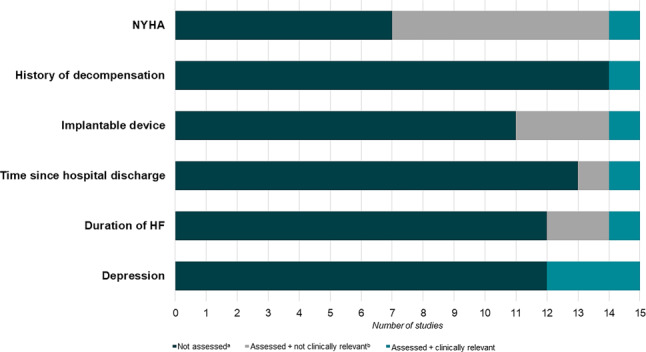
Clinically relevant subgroups. The effect of telemedicine on mortality and (re)hospitalisation was heterogeneous across these six patient characteristics. These characteristics were not examined in all of the included trials. Among the studies in which the characteristic was assessed, some found clinically relevant (significant) outcomes, while others did not find significant effects. *NYHA* New York Heart Association, *HF* heart failure; ^a^(Not) assessed = characteristic has (not) been examined in a study; ^b^(Not) clinically relevant = telemedicine and characteristics have a positive or negative effect on mortality and/or (re)hospitalisation

From the RCTs included, we extracted 21 different subgroups related to the characteristics of HF patients (Tab. 1). Overall, the methodology of the subgroup analyses performed was not optimal. Subgroup definitions were inconsistent, not specified a priori [10–13] and the motivation for subgroup analysis was not always reported [14–24]. Many subgroups comprised a limited number of individuals, and sample size calculations did not specifically target subgroup analyses [10–24]. In summary, the effect of telemedicine on mortality and (re)hospitalisation was heterogeneous across six patient characteristics: NYHA classification, previous HF decompensation, having an implantable device, time since hospital discharge for HF, duration of HF and concurrent depression (Fig. 3; [11, 17, 20, 23, 24]). We discuss the findings on subgroup analyses in detail below.

**Table 1:** Number of randomised controlled trials that reported subgroup analyses related to patient characteristics

Categories of patient characteristics:	
Demographics	Age (*n* = 8), sex (*n* = 8), ethnicity (*n* = 3)
HF characteristics	Aetiology of HF (*n* = 4), LVEF (*n* = 8), NYHA class (*n* = 8), history of decompensation (*n* = 1), implantable device (*n* = 4), previous hospitalisation (*n* = 2), duration of HF (*n* = 3)
Multimorbidity	Atrial fibrillation (*n* = 2), heart rate (≤ 70 or > 70 beats/min) (*n* = 1), depression (*n* = 3)
Social network	Living alone (*n* = 1), living environment (rural/urban) (*n* = 1), involvement of an informal caregiver (*n* = 1)
Laboratory values	NT-proBNP (*n* = 5), MR-proADM (*n* = 2), MR-proANP (*n* = 1), eGFR (*n* = 3)
Medication prescription	Dose of diuretics (*n* = 1)

*HF* heart failure, *LVEF* left ventricular ejection fraction, *NYHA* New York Heart Association, *NT-proBNP* N-terminal pro B‑type natriuretic peptide, *MR-proADM* mid-regional pro-adrenomedullin, *MR-proANP* mid-regional pro-atrial natriuretic peptide*, eGFR* estimated glomerular filtration rate

### Demographics

Nine trials studied the effects of telemedicine on (re)hospitalisation and mortality in subgroups based on: age [10, 12–15, 17, 21, 24] sex [10, 12–15, 17, 21, 24] and ethnicity [10, 14, 18]. No differences in the effects of telemedicine were found across these subgroups.

### Heart failure characteristics

Fourteen trials studied the effects of telemedicine on (re)hospitalisation and mortality in subpopulations related to the following HF characteristics: aetiology of HF [12, 15, 22, 24], left ventricle ejection fraction (LVEF) [11–17, 24], NYHA classification [10, 11, 13, 14, 17, 18, 21, 23], history of HF decompensation [17], implantable device [11, 13, 17, 21], previous HF hospitalisation [12, 24] and duration of HF diagnosis [11, 12, 19].

No differences in the effects of telemedicine were found across HF aetiology (ischaemic/non-ischaemic) or LVEF. The definition of subgroups based on LVEF was heterogeneous; six of eight trials employed varying cut-off values for the dichotomous variable (Table S1 (Electronic Supplementary Material)). The data-driven defined cut-off values diverged from established HF guidelines [25, 26].

Only one out of eight trials assessed heterogeneity in telemedicine effectiveness across NYHA classification. This study found that telemedicine was slightly more protective in the NYHA III/IV group compared to the total study population [23]. The hazard ratio (HR) expressing the effect of telemedicine versus usual care on first unplanned hospitalisation was 0.71 [95% confidence interval (CI) 0.53–0.95] in the NYHA III/IV group and 0.79 (95% CI 0.62–0.99) in the total study population.

One study assessed heterogeneity in the effect of telemedicine on mortality across subgroups with and without episodes of decompensation prior to randomisation [17]. This study found that in patients with episodes of decompensation, the use of telemedicine was not associated with mortality during the study follow-up with an HR of 0.87 (95% CI 0.58–1.30) compared to usual care. This HR was 2.23 (95% CI 0.69–7.25) in patients without episodes of decompensation. Replication of this analysis would help to further understand this finding, as the number of events was low, particularly in the group without episodes of decompensation (4 and 9 events, respectively).

Only one out of four trials assessing heterogeneity in telemedicine effectiveness across implantable device status found a statistically significant heterogeneity in effect. This study found that in patients with a pacemaker, the use of telemedicine reduced the 1‑year risk of HF-related hospitalisations with an HR of 0.37 (95% CI 0.15–0.93) compared to usual care, while in patients without a pacemaker this HR was 0.96 (95% CI 0.42–2.21) [11]. We speculate this might be due to chance, given that the number of events in the subgroups was low (7 and 14 events, respectively) and the other three trials did not find this effect.

One study assessed subgroups with varied time between discharge after HF hospitalisation and the start of telemedicine use (discharged ≤ 30 days vs > 30 days before enrolment) [24]. This study found that in patients discharged ≤ 30 days before enrolment using telemedicine compared to usual care increased the risk of HF-related hospitalisation or cardiovascular death with an HR of 1.05 (95% CI 0.64–1.72), while for patients discharged > 30 days before enrolment this HR was 0.48 (95% CI 0.27–0.84). Further research into the optimal start of telemedicine could clarify whether there is true effect heterogeneity or another explanation for the difference in effect.

Only one out of three trials assessing heterogeneity in telemedicine effectiveness across the duration of HF diagnosis found a statistically significant heterogeneity in effect [11]. In patients with an HF history ≤ 18 months using telemedicine compared to usual care the 1‑year risk of HF-related hospitalisations was reduced with an HR of 0.26 (95% CI 0.07–0.94), while this HR was 0.89 (95% CI 0.42–1.85) in patients with an HF history > 18 months. Again, replication of this analysis would help to further understand the credibility of this evidence, as the number of events in these subgroups was low (3–15 events).

### Multimorbidity

Five trials studied the effects of telemedicine on (re)hospitalisation and mortality in subpopulations related to the following multimorbidities: atrial fibrillation (AF) [11, 12], heart rate [11], depression [17, 20], social isolation defined by the 36-item Short Form Health Survey (SF-36) mental health score [23].

Trials investigating the effect of telemedicine on (re)hospitalisation and mortality in subgroups based on the presence of AF showed no differences in effect [11, 12]. One study performed subgroup analyses based on heart rate (≤ 70 beats/min or > 70 beats/min) and found no heterogeneity of the effect of telemedicine on a composite of HF hospital admission and all-cause mortality [11].

Two publications from the same RCT studied the effectiveness of telemedicine on (re)hospitalisation and mortality in patients with and without depression [17, 20]. Pre-specified subgroup analysis for depression [Patient Health Questionnaire (PHQ-9) score ≥ 10 points] showed no differences in the effectiveness of telemedicine on the primary outcome ‘mortality’ but found a difference for the secondary outcome ‘days lost to HF hospitalisation or death’. Telemedicine seemed harmful in the subgroup with depression (mean days lost to HF-related hospitalisation or death ± SE: 49.4 ± 10.1 for telemedicine use vs 29.1 ± 10.2 for usual care), and telemedicine was effective in the subgroup without depression (mean days lost to HF-related hospitalisation or death ± SE: 27.8 ± 5.5 for telemedicine use vs 42.0 ± 5.5 for usual care) [17]. The second publication focused on an additional secondary outcome ‘improvement of depression’ and found that telemedicine improved 1‑year depression PHQ‑9 score in patients with a depression at baseline [adjusted mean difference of −1.6 (95% CI −2.4 to −0.7) for telemedicine users and −0.2 (95% CI −1.1 to 0.7) for usual care] but did not influence the 1‑year PHQ‑9 score in patients without baseline depression [20].

One study found telemedicine to reduce the number of deaths and unplanned hospitalisations (composite) at 18 months by around half an event on average in socially isolated patients [from a mean of 1.3 (SD ± 1.7) to 1.9 (SD ± 2.1)] [23]. Results were not reported for patients who were not socially isolated.

### Social network

Three trials studied the impact of social network: living alone versus co-habiting [11], living environment rural versus urban [13], and presence or absence of an informal caregiver [19]. No differences in the effects of telemedicine on hospitalisation or mortality were found across variations of patients’ social network.

### Laboratory values and medication prescription

Seven trials studied the effects of telemedicine on (re)hospitalisation and mortality in subgroups based on baseline laboratory variables: N‑terminal pro-B-type natriuretic peptide [13, 15–17, 19], mid-regional pro-adrenomedullin [13, 17], mid-regional pro-atrial natriuretic peptide [17], estimated glomerular filtration rate [13, 17, 24] and HF medication (dose of diuretics) [15]. No differences in effects of telemedicine were found across subgroups. However, in all studies, continuous laboratory measures were categorised, reducing the power to detect heterogeneity by that variable (Table S1 (Electronic Supplementary Material)).

## Discussion and future directions

Ideally, medical decisions about the use of telemedicine in specific HF patients rely on solid scientific evidence. This review reveals inconsistent evidence on the groups that benefit most, complicating decisions on targeted telemedicine provision (i.e. patient selection, moment in HF trajectory). A small number of studies found that some patient subgroups receiving telemedicine have slightly better outcomes: patients with NYHA III/IV, who have experienced HF decompensation, who started telemedicine > 30 days after hospital discharge or were diagnosed with HF for ≤ 18 months. Notably, we did not find an RCT comparing patients with different SES levels and the effectivity of telemedicine on (re)hospitalisation and mortality. However, it is known that patients with lower SES are at higher risk of morbidity and mortality [27], and these patients have lower adoption rates of telemedicine and fewer benefits therefrom [28]. It seems relevant that future studies should collect data on SES and evaluate heterogeneity of telemedicine effectiveness across SES status.

The findings regarding effect modifiers should be read in the context of the participants actually represented in RCTs. The eligibility criteria and baseline characteristics illustrate that it is challenging to apply these results to patients with multiple heart diseases, CKD, cognitive and physical problems, as well as to those living in a nursing home and having a life expectancy less than 1 year. It is noteworthy that cognitive problems are common in HF patients, ranging between 25% and −75%, whereas these patients are mostly not eligible to participate in RCTs [29]. In addition, the prevalence of concurrent diabetes and depression in the general HF population is higher compared to that in the trial populations [10, 12–15, 17–24, 29, 30]. This implies that findings on effectiveness of telemedicine from RCTs might not be generalisable to all HF patients.

### Future directions

#### Evidence on ‘in whom’

Future studies on telemedicine’s impact on (re)hospitalisation in heterogeneous HF populations should broaden inclusion criteria for better representation. To move beyond exploratory evaluations of treatment effect heterogeneity, sample size calculations should aim for subgroup analyses [31]. While subgroup analyses provide insights into treatment heterogeneity across a single variable (e.g. males versus females), the clinical interest is often in effects for subgroups that are defined in a multivariable way (e.g. males with/without AF vs females with/without AF). Such an in-depth analysis of treatment heterogeneity can be conducted in accordance with the PATH statement [32]. This statement explains how to stratify individuals into subgroups using a prognostic model for the outcome of interest and, subsequently, assess treatment heterogeneity across these subgroups in RCTs. To implement this, studies should be set up to derive and externally validate a prognostic model that predicts risk of (re)hospitalisation in an HF population at the time of telemedicine initiation [33, 34]. Current research makes it difficult to formulate which patient characteristics (single or multivariable) need most evidence, since the scoping review reveals a paucity of evidence across all patient characteristics. Engaging the healthcare professionals is essential to select important subgroups which need be researched first. As a starting point, focusing on fundamental patient and HF characteristics, such as sex, age, SES, HF duration since diagnosis, recent exacerbations, NYHA classification, and comorbidities such as depression, could provide valuable insights.

#### Evidence on ‘when’

Telemedicine can be initiated at multiple phases in the course of HF: after diagnosis, during medication optimisation (titration phase), after HF decompensation for stabilisation, and even during the palliative phase to explore its added value. Tailoring the intervention components (frequency of monitoring, self-care modules, education) can be of added value in these phases. Future studies must carefully define the research question and explicitly state the study’s temporal origin, which indicates when an individual becomes at risk for the outcome and eligible for telemedicine [35]. While the ultimate interest is in understanding telemedicine’s effect on (re)hospitalisation across all HF phases, establishing a unique temporal origin in a (sub)analysis (for example 1 month after rehospitalisation or during the titration phase), which aligns with healthcare professionals needs, facilitates clear interpretation of findings [36]. The analysis should ensure comparability of intervention groups at the temporal origin to prevent confounding bias [37] and allocate person-time to avoid time-related biases [38].

#### Increasing credibility of evidence

Estimates of telemedicine effectiveness are not always consistent since studies differ in included populations and implementation (and timing) of the telemedicine intervention. This requires formulating more specific research questions to make clear what intervention is studied in whom and when [39].

Another approach to ascertain the credibility of findings is through replication studies. These can involve repeating the study analysis in new data to assess the similarity of results to the original (direct replication) [40]. Alternatively, conceptual replication can be conducted to assess the generalisability of earlier findings, such as emulating an RCT using observational data [41].

#### Observational studies to establish (causal) evidence

Most evidence on telemedicine effectiveness is currently derived from RCTs, while the widespread roll-out of telemedicine in clinical practice allows for observational studies using real-world health data, like electronic health records (EHRs) linked with device data. Investigating causal effects using observational data poses challenges [42]. Causal analyses using observational data involve additional assumptions, with the absence of confounding being a key consideration through adjustment for confounding variables. Confounding variables should be identified, using literature, before analysing the data [43, 44]. While many prognostic factors for (re)hospitalisation and other outcomes are known, factors influencing the decision to start telemedicine are less well understood. To enhance confounding adjustment in observational studies, studying how telemedicine is assigned is valuable; crucial aspects are the registration of the indication to start telemedicine and documenting patient preferences.

#### Learning healthcare systems: updating evidence in clinical practice

**Fig. 4:**
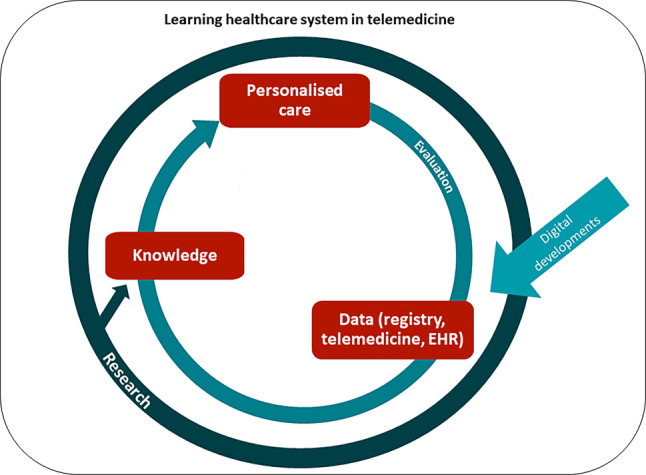
A learning healthcare system in heart failure management using telemedicine is an iterative process: evaluating current treatment of the patient and data from the electronic health record (*EHR*). (Continuously developing) telemedicine devices are used to inform healthcare professionals about the health status of the patient. This information, combined with scientific research findings, leads to knowledge that supports clinical decision making, resulting in personalised treatment. These personalised treatments are then re-evaluated

Another complexity in evaluating telemedicine effects stems from its dynamic and evolving nature, characterised by a high turnover of new technologies and components. Besides, heterogeneity in telemedicine interventions and diverse patient populations adds complexity. Traditional research designs like RCTs are deemed less appropriate and too costly [45, 46]. Therefore, staying abreast of new technologies and evaluating their impact requires methodological innovation, such as utilising real-world health data. A promising concept to identify ‘what works best for whom’ in a data-intensive domain is a learning healthcare system (LHS) [47]. An LHS consists of the components care, data and evidence, which form a cycle that accelerates evidence generation in a rapidly changing environment, improve care and inform professionals (Fig. 4; [48]). In the context of telemedicine research, an LHS allows real-time analysis of EHR data complemented with telemonitoring data, providing insights into the comparative effectiveness of telemedicine treatments and surveillance of adverse events. This supports clinicians in treatment decisions and facilitates personalised treatment [49]. Databases specific to HF can be utilised, e.g, for the Dutch setting the HF registry of the Netherlands Heart Registration (NHR-HF registry) [50]. Collaboration between healthcare professionals, telemedicine providers and epidemiologists is important. An initial LHS in the field of HF and telemedicine is planned to be developed by the RELEASE-HF study [51], aiming to empower the healthcare provider to apply telemedicine in a more effectively targeted way, considering patient characteristics and the innovating environment of telemedicine.

## Conclusion

This review highlights the absence of definite scientific evidence regarding which HF patients benefit most from telemedicine. The lack of specific guidance poses a dilemma for clinicians: prescribing telemedicine to all patients or targeting telemedicine to specific subgroups of patients. Future research should delve into the heterogeneous effectiveness of telemedicine across patient subgroups, identifying when and for whom it proves most beneficial during the course of HF, but also how telemedicine can be tailored at the individual level, resulting in a dynamic use of telemedicine components appropriate to a periodic need of the patient. Specifically, studies are needed to explore meaningful subgroup establishment that investigate through prognostic modelling techniques and determine optimal timing of telemedicine in the HF trajectory. Evidence on effectiveness can be established in studies that calculate the required sample size with the specific goal of subgroup analyses in mind. Such principled studies can eventually form the basis for dynamically updated evidence, i.e. LHS. The LHS using real-world health data and telemonitoring data could facilitate ongoing monitoring to identify who benefits from telemedicine at various points in the HF trajectory.

### Supplementary Information

Table S1: Study characteristics of the included randomised controlled trials

Table S2: Overview of meta-analyses (*n* = 19) used for the selection of randomized controlled trials
