# Supplementary material for: Which heart failure patients benefit most from non-invasive telemedicine? An overview of current evidence and future directions
Source: Neth Heart J. 2024 Aug 14;32(9):304–14. doi: 10.1007/s12471-024-01886-4 (PMC11336005; doi:10.1007/s12471-024-01886-4)
Supplement: Supplementary file 1 — Table S1: Study characteristics of the included randomised controlled trials [file 12471_2024_1886_MOESM1_ESM.docx]

**Table 1** Study characteristics of the included randomised controlled trials: study population, non-invasive telemedicine intervention, outcome, definition of performed subgroup analyses

| Study (year) | Population | Intervention | Control | Outcome (primary) | Subgroup definitions |
| --- | --- | --- | --- | --- | --- |
| AMULET [24]  (2022) | Patients with HF and LVEF ≤49%, after an episode of acute HF within the last 6 months | Seven nurse-led assessments, transmission of recorded parameters and clinical features to telemedicine website service with presentation within recommendation support module, cardiologist makes remote therapeutic decisions | Patients remain under supervision of cardiologist and other physicians using the facilities available in the usual healthcare system based on current clinical needs | Composite of first unplanned HF hospitalisation and/or cardiovascular death in 12 months of follow-up | Age (years): <65 or ≥65  Sex: female or male  Ischaemic aetiology: no or yes  LVEF: <40% or 40-49%  Discharge time (days): ≤30 or >30  eGRF (ml/min per 1.73 m^2^): <60 or ≥60 |
| TIM-HF [20]  (2021) | Ambulatory patients with stable HF who receive optimal medical treatment. NYHA class II or III and LVEF ≤25% measured at least twice within the past 6 months or LVEF ≤35% and at least one cardiac decompensation with hospitalisation due to HF or therapy with intravenous diuretics within 24 months prior to enrolment | Remote monitoring data sent daily to central location, STS, 24/7 physician-led call centre | Guideline-based care in HF (NYHA II-III) including at least 5 scheduled doctor's visits | Change from baseline in depression score at 12 months, measured using the PHQ-9 questionnaire | PHQ-9 score: <10 (non-depressed) or ≥10 (depressed) |
| TELEREH-HF [22]  (2021) | Stable HF patients (NYHA I, II, or III) who had LVEF ≤ 40% after a hospitalisation, within 6 months prior to the randomisation | 9-week hybrid comprehensive telerehabilitation, week 1 hospital, week 2-9 home 5× weekly | Baseline clinical examinations during a 3-day hospitalisation and 9-week observation. Usual care according to guidelines. Rehabilitation or remote monitoring via CIEDS is possible. Final assessment after 9th week, during 3-day hospitalisation. Recommendations for lifestyle changes and self-management according to guidelines | Percentage of days alive and out of hospital during 14-26 months of follow-up | Aetiology: ischaemic or non-ischaemic |
| OSICAT [23]  (2020) | Patients hospitalised for acute HF ≤12 months before inclusion | Measured body weight sent daily, symptom questions answered daily, nurse-controlled, alerts, contact on working days. Personalised education, telephone call every 3 weeks | Conventional follow-up at the discretion of the patient’s GP or referring cardiologist | Composite of all-cause death and/or unplanned hospitalisation at 18 months | NYHA: III or IV  Socially isolated patient (defined as: SF-36 Mental Health score <45 or Mental Component Summary score <35 or Mental Health <50 plus Mental Component Summary <40 or medical history of depression/mood disorders/alterations or use of concomitant antidepressant medications): yes or no |
| TELEREH-HF [21]  (2020) | Stable HF patients (NYHA I, II, or III) who had LVEF ≤ 40% after a hospitalisation, within 6 months prior to the randomisation | 9-week hybrid comprehensive telerehabilitation, week 1 hospital, week 2-9 home 5× weekly | Baseline clinical examinations during a 3-day hospitalisation and 9-week observation. Usual care according to guidelines. Rehabilitation or remote monitoring via CIEDS possible. Final assessment after 9th week, during 3-day hospitalisation. Recommendations for lifestyle changes and self-management according to guidelines | Percentage of days alive and out of hospital during 14-26 months of follow-up | Age (values not specified)  Sex: female or male  NYHA: I + II or III  CIEDS: yes or no |
| REACH-HF [19]  (2019) | HF patients with LVEF ≤45% | 12-week telephone and face-to-face contact, exercise ≥3 times per week, progress tracker, family resource and cardiac nurse or physiotherapist support | No cardiac rehabilitation approach that included medical management according to national and local guidelines, including specialist HF nurse care | Disease-specific HRQoL at 12 months measured using the Minnesota Living with Heart Failure Questionnaire | Time since diagnosis of HF (years): <1 or 1-2 or >2  Caregiver present: yes or no  NT-proBNP (pg/ml): ≤2000 or >2000 |
| Pekmezaris et al. [18]  (2019) | Black and Hispanic patients from underserved communities with a primary diagnosis of HF, NYHA I, II or III and no cognitive problems | Daily self-monitoring of vital signs, weekly telehealth visits by nurse, assessment of vital signs during office hours. Monitoring, education, visits every 3 months | One clinic visit after discharge, weekly ‘check-in’ phone calls during 1st month. HF clinic visit or phone-based clinician assessment if worsening HF or major weight changes. Usual follow-up by patient’s cardiologist or HF clinic. | Number of emergency department visits, inpatient utilisation, and length of stay over a 90-day period. | Ethnicity: Hispanic or Black  NYHA: II or III |
| TIM-HF2 [13]  (2018) | Patients with HF, NYHA II or III, admitted to hospital for HF within 12 months before randomisation, LVEF of ≤45% | Remote monitoring data sent daily to central location, STS, 24/7 physician-led call centre | Follow-up in accordance with current guidelines for management and treatment of patients with HF | Percentage of days lost due to unplanned cardiovascular hospitalisation or all-cause mortality | Age (years): ≤73 or >73  Sex: female or male  LVEF: ≤45% or >45%  NYHA: I or II or III or IV  Device: CRT, yes or no  ICD yes or no  Living environment: rural or urban  MR-proADM (nmol/l): <1.2 or >1.2  NT-proBNP (pg/ml) <901 or 901-2250 or >2250  GFR (ml/min per 1.73 m^2^): <30 or 30-60 or >60 |
| BEAT-HF [10]  (2016) | Patients (≥50 years) admitted as hospital inpatients or with observation status, receiving active treatment for decompensated HF | Education, telephone coaching, remote measurement data sent daily to nurse-led call centre | Robust pre-discharge education and often a post-discharge follow-up telephone call. No additional surveillance provided beyond whatever may have been requested as part of routine clinical practice | 180-day all-cause readmission | Age (years): ≤65 or >65  Sex: female or male  Ethnicity: African American or Hispanic/Latino or White or Asian/Pacific islander or other  NYHA: not III/IV or III/IV |
| WISH [12]  (2014) | Patient hospitalised for HF, NYHA III-IV, LVEF <50%. Treatment with diuretics or other HF medication | Daily self-weight control. Weight automatically sent to system, checked by nurses (office hours). Using alarms. Weight gain leads to telephone contact and follow-up questions | Daily weight control, if weight gain >2 kg in 3 days contact (by patient) with HF clinic | Time to cardiac re-hospitalisation | Age (years): <75 or ≥75  Sex: female or male  Aetiology: ischaemic or non-ischaemic  LVEF: ≤25% or >25%  Previous hospitalisation: yes or no  Previous HF: yes or no  AF: sinus rhythm or AF |
| TEHAF [11]  (2012) | Patients with HF, NYHA II-IV, LVEF ≤40% or a preserved ejection fraction with diastolic dysfunction | Device with four keys, heart rate and blood pressure collected via face-to-face contacts. Daily pre-set HF dialogues, daily questions about HF. Answers to questions lead to a risk profile with different intensity of monitoring and education | Nurse-led usual care according to latest European Society of Cardiology guideline. Regular face-to-face contact for evaluation of heart rate and blood pressure | Time to first HF hospitalisation | LVEF: ≤45% or >45%  NYHA: II or III or IV  HF duration (months): ≤18 or >18  Device: pacemaker, yes or no  AF: yes or no  Charlson index: ≤2 or >2  Living alone: yes or no |
| TEMA-HF [16]  (2012) | Patients hospitalised for HF. Treated with HF medication | 1 h HF education course, 6 months telemonitoring, Bluetooth-connected systems, alerts, website for communication between patient and HF nurse, planned follow-up at 3 and 6 months | Two weeks after discharge, evaluation of fluid status and optimisation of treatment by outpatient HF clinic. Follow-up by GP, who could refer patients to their cardiologist | 180-day all-cause mortality | LVEF: <30% or ≥30%  NT-proBNP (values not specified) |
| TIM-HF [17]  (2012) | Ambulatory patients with stable HF, receiving optimal medical treatment according to current guidelines. NYHA class II or III and LVEF ≤25% measured at least twice within the past 6 months or LVEF ≤35% and at least one cardiac decompensation with hospitalisation due to HF or therapy with intravenous diuretics within 24 months prior to enrolment | Remote monitoring data sent daily to central location, STS, 24/7 physician-led call centre | Guideline-based care in HF (NYHA II-III), including at least 5 scheduled doctor's visits | All-cause mortality during an unspecified time period (median follow-up time of 26 months in the study sample) | Age (years): <70 or ≥70  LVEF: <27% or ≥27%  NYHA: II or III  Decompensation history: yes or no  Device: ICD, yes or no  Depression: PHQ-9 score; <10 or ≥10  NT-proBNP (pg/ml): <2089 or ≥2089  MR-proADM (nmol/l): <1.05 or ≥1.05  MR-proANP (pmol/l): <371.5 or ≥371.5  eGFR (ml/min): <60 or ≥60 |
| TELE-HF [14]  (2010) | Patient hospitalised for HF in previous 30 days | Educational materials and a weight scale. Daily remote monitoring data and phone calls with responses to automated questions | Educational materials and a weight scale | Composite of all-cause readmissions and/or all-cause mortality within 180 days | Age (years): <65 or ≥65  Sex: female or male  Ethnicity: White or Black or other  LVEF: <40% or ≥40%  NYHA: I/II or III/IV |
| TEN-HMS [15]  (2005) | Patients with a recent admission for HF and LVEF <40% | *Telemonitoring (intervention 1)*:  Remote monitoring data sent twice daily to central location, nurse telephone support each month by HF specialist to assess symptoms and current medication  *Telephone support (intervention 2)*:  Nurse telephone support each month by HF specialist to assess symptoms and current medication | Pharmacological treatment plan implemented by primary care physician. Patients assessed at a research clinic every 4 months to assess intervening history, symptoms and signs, renal function and serum electrolytes. Contact with the research team discouraged between visits | Days lost because of death or hospitalisation during 240 days | Age (values not specified)  Sex: female or male  LVEF (values not specified)  Aetiology: ischaemic or other  Dose of diuretics (mg/day): 40 or >40  NT-proBNP (values not specified) |
| *HF* heart failure, *LVEF* left ventricle ejection fraction, *eGRF* estimated glomerular filtration rate, *NYHA* New York Heart Association, *CIEDS* cardiovascular implantable electronic devices, *STS* structured telephone support, *PHQ-9* Patient Health Questionnaire-9, *HRQoL* health-related quality of life, *NT-proBNP* N-terminal pro-B-type natriuretic peptide, *MR-proADM* mid-regional pro-adrenomedullin, *MR-proANP* mid-regional pro-atrial natriuretic peptide, *CRT* cardiac resynchronisation therapy, *ICD* implantable cardioverter-defibrillator, *AF* atrial fibrillation, *GP* general practitioner | | | | | |
