# Supplementary material for: Which heart failure patients benefit most from non-invasive telemedicine? An overview of current evidence and future directions
Source: Neth Heart J. 2024 Aug 14;32(9):304–14. doi: 10.1007/s12471-024-01886-4 (PMC11336005; doi:10.1007/s12471-024-01886-4)
Supplement: Supplementary file 2 — Table S2: Overview of meta-analyses (n = 19) used for the selection of randomized controlled trials [file 12471_2024_1886_MOESM2_ESM.docx]

**Supplemental 1**

**Table S1.** Overview of meta-analyses (n=19) used for the selection of randomized controlled trials.

| **Author, year** | **Title** | **Number of trials** |
| --- | --- | --- |
| Scholte et al., 2023^1^ | Telemonitoring for heart failure: a meta-analysis. | Total: 92  Non-invasive trials: 65 |
| Rebolledo Del Toro et al., 2023^2^ | Effectiveness of mobile telemonitoring applications in heart failure patients: systematic review of literature and meta-analysis | Non-invasive trials: 19 |
| Liu et al., 2022^3^ | Effectiveness of eHealth Self-management Interventions in Patients With Heart Failure: Systematic Review and Meta-analysis | Non-invasive trials: 24 |
| Chauhan et al., 2022^4^ | Comparison of Mortality and Hospital Readmissions Among Patients Receiving Virtual Ward Transitional Care vs Usual Postdischarge Care: A Systematic Review and Meta-analysis | Total: 24  Heart failure population trials: 10 |
| Mhanna et al., 2022^5^ | Efficacy of remote physiological monitoring-guided care for chronic heart failure: an updated meta-analysis | Total: 16 |
| Kitsiou et al., 2021^6^ | Effectiveness of mobile health technology interventions for patients with heart failure: systematic review and meta-analysis. | Total: 16 |
| Ma et al., 2021^7^ | The efficacy of telemedical care for heart failure: A meta-analysis of randomized controlled trials | Total: 4 |
| Amirova et al., 2021^8^ | Efficacy of interventions to increase physical activity for people with heart failure: a meta-analysis | Total: 20 |
| Drews et al., 2021^9^ | Non-invasive home telemonitoring in patients with decompensated heart failure: a systematic review and meta-analysis | Total: 11 |
| Cassidy et al., 2021^10^ | The impact of psychoeducational interventions on the outcomes of caregivers of patients with heart failure: A systematic review and meta-analysis. | Total: 10 |
| Indraratna et al., 2020^11^ | Mobile phone technologies in the management of ischemic heart disease, heart failure, and hypertension: systematic review and meta-analysis | Total: 26  Heart failure population trials: 6 |
| Zhu et al., 2020^12^ | Effectiveness of telemedicine systems for adults with heart failure: a meta-analysis of randomized controlled trials | Total: 29 |
| Ding et al., 2020^13^ | Effects of Different Telemonitoring Strategies on Chronic Heart Failure Care: Systematic Review and Subgroup Meta-Analysis | Total: 26 |
| Kraef et al., 2020^14^ | Digital telemedicine interventions for patients with multimorbidity: a systematic review and meta-analysis | Total: 5 |
| Son et al., 2020^15^ | Effectiveness of Mobile Phone-Based Interventions for Improving Health Outcomes in Patients with Chronic Heart Failure: A Systematic Review and Meta-Analysis | Total: 8 |
| Oldridge et al., 2019^16^ | A systematic review of recent cardiac rehabilitation meta-analyses in patients with coronary heart disease or heart failure | Total: 30  Heart failure population trials: 12 |
| Aronow et al., 2018^17^ | Comparative Effectiveness of Disease Management With Information Communication Technology for Preventing Hospitalization and Readmission in Adults With Chronic Congestive Heart Failure | Total: 58 |
| Pekmezaris et al., 2018^18^ | Home telemonitoring in heart failure: a systematic review and meta-analysis | Total: 26 |
| Yun et al., 2018^19^ | Comparative Effectiveness of Telemonitoring Versus Usual Care for Heart Failure: A Systematic Review and Meta-analysis | Total: 37 |

**References**

1. Scholte NT, Gürgöze MT, Aydin D*, et al.* Telemonitoring for heart failure: a meta-analysis. *European Heart Journal* 2023:ehad280. doi:

2. Rebolledo Del Toro M, Herrera Leano NM, Barahona-Correa JE, Munoz Velandia OM, Fernandez Avila DG, Garcia Pena AA. Effectiveness of mobile telemonitoring applications in heart failure patients: systematic review of literature and meta-analysis. *Heart Fail Rev* 2023;**28**:431-452. doi: 10.1007/s10741-022-10291-1

3. Liu S, Li J, Wan DY*, et al.* Effectiveness of eHealth Self-management Interventions in Patients With Heart Failure: Systematic Review and Meta-analysis. *J Med Internet Res* 2022;**24**:e38697. doi: 10.2196/38697

4. Chauhan U, McAlister FA. Comparison of Mortality and Hospital Readmissions Among Patients Receiving Virtual Ward Transitional Care vs Usual Postdischarge Care: A Systematic Review and Meta-analysis. *JAMA Netw Open* 2022;**5**:e2219113. doi: 10.1001/jamanetworkopen.2022.19113

5. Mhanna M, Beran A, Nazir S*, et al.* Efficacy of remote physiological monitoring-guided care for chronic heart failure: an updated meta-analysis. *Heart Fail Rev* 2022;**27**:1627-1637. doi: 10.1007/s10741-021-10176-9

6. Kitsiou S, Vatani H, Paré G*, et al.* Effectiveness of mobile health technology interventions for patients with heart failure: systematic review and meta-analysis. *Canadian Journal of Cardiology* 2021;**37**:1248-1259. doi:

7. Ma X, Li J, Ren X. The efficacy of telemedical care for heart failure: A meta-analysis of randomized controlled trials. *Am J Emerg Med* 2021;**47**:1-5. doi: 10.1016/j.ajem.2021.01.032

8. Amirova A, Fteropoulli T, Williams P, Haddad M. Efficacy of interventions to increase physical activity for people with heart failure: a meta-analysis. *Open Heart* 2021;**8**. doi: 10.1136/openhrt-2021-001687

9. Drews TEI, Laukkanen J, Nieminen T. Non-invasive home telemonitoring in patients with decompensated heart failure: a systematic review and meta-analysis. *ESC Heart Fail* 2021;**8**:3696-3708. doi: 10.1002/ehf2.13475

10. Cassidy L, Hill L, Fitzsimons D, McGaughey J. The impact of psychoeducational interventions on the outcomes of caregivers of patients with heart failure: A systematic review and meta-analysis. *Int J Nurs Stud* 2021;**114**:103806. doi: 10.1016/j.ijnurstu.2020.103806

11. Indraratna P, Tardo D, Yu J*, et al.* Mobile phone technologies in the management of ischemic heart disease, heart failure, and hypertension: systematic review and meta-analysis. *JMIR mHealth and uHealth* 2020;**8**:e16695. doi:

12. Zhu Y, Gu X, Xu C. Effectiveness of telemedicine systems for adults with heart failure: a meta-analysis of randomized controlled trials. *Heart failure reviews* 2020;**25**:231-243. doi:

13. Ding H, Chen SH, Edwards I*, et al.* Effects of Different Telemonitoring Strategies on Chronic Heart Failure Care: Systematic Review and Subgroup Meta-Analysis. *J Med Internet Res* 2020;**22**:e20032. doi: 10.2196/20032

14. Kraef C, van der Meirschen M, Free C. Digital telemedicine interventions for patients with multimorbidity: a systematic review and meta-analysis. *BMJ Open* 2020;**10**:e036904. doi: 10.1136/bmjopen-2020-036904

15. Son YJ, Lee Y, Lee HJ. Effectiveness of Mobile Phone-Based Interventions for Improving Health Outcomes in Patients with Chronic Heart Failure: A Systematic Review and Meta-Analysis. *Int J Environ Res Public Health* 2020;**17**. doi: 10.3390/ijerph17051749

16. Oldridge N, Pakosh M, Grace SL. A systematic review of recent cardiac rehabilitation meta-analyses in patients with coronary heart disease or heart failure. *Future Cardiol* 2019;**15**:227-249. doi: 10.2217/fca-2018-0085

17. Aronow WS, Shamliyan TA. Comparative Effectiveness of Disease Management With Information Communication Technology for Preventing Hospitalization and Readmission in Adults With Chronic Congestive Heart Failure. *J Am Med Dir Assoc* 2018;**19**:472-479. doi: 10.1016/j.jamda.2018.03.012

18. Pekmezaris R, Tortez L, Williams M*, et al.* Home telemonitoring in heart failure: a systematic review and meta-analysis. *Health Affairs* 2018;**37**:1983-1989. doi:

19. Yun JE, Park JE, Park HY, Lee HY, Park DA. Comparative Effectiveness of Telemonitoring Versus Usual Care for Heart Failure: A Systematic Review and Meta-analysis. *J Card Fail* 2018;**24**:19-28. doi: 10.1016/j.cardfail.2017.09.006
